# Supplementary material for: Collective excitation of plasmon-coupled Au-nanochain boosts photocatalytic hydrogen evolution of semiconductor
Source: Nat Commun. 2019 Oct 29;10:4912. doi: 10.1038/s41467-019-12853-8 (PMC6820756; doi:10.1038/s41467-019-12853-8)
Supplement: Supplementary file 1 — Supplementary Information [file 41467_2019_12853_MOESM1_ESM.pdf]

Supplementary Information

Collective excitation of plasmon-coupled Au-nanochain boosts photocatalytic hydrogen evolution of semiconductor

Yu *et al.*

Supplementary Figures

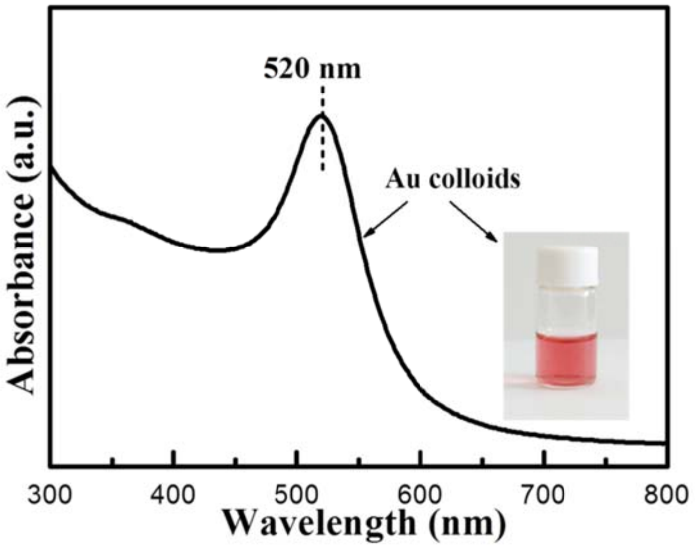

Supplementary Figure 1| UV-vis absorption spectrum and photograph (inset) of Au colloids.

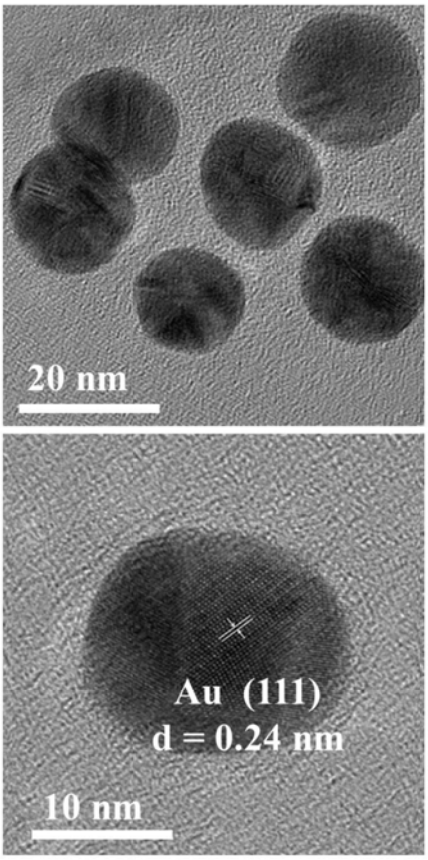

Supplementary Figure 2| TEM images of Au colloids prepared with a sodium citrate reduction method.

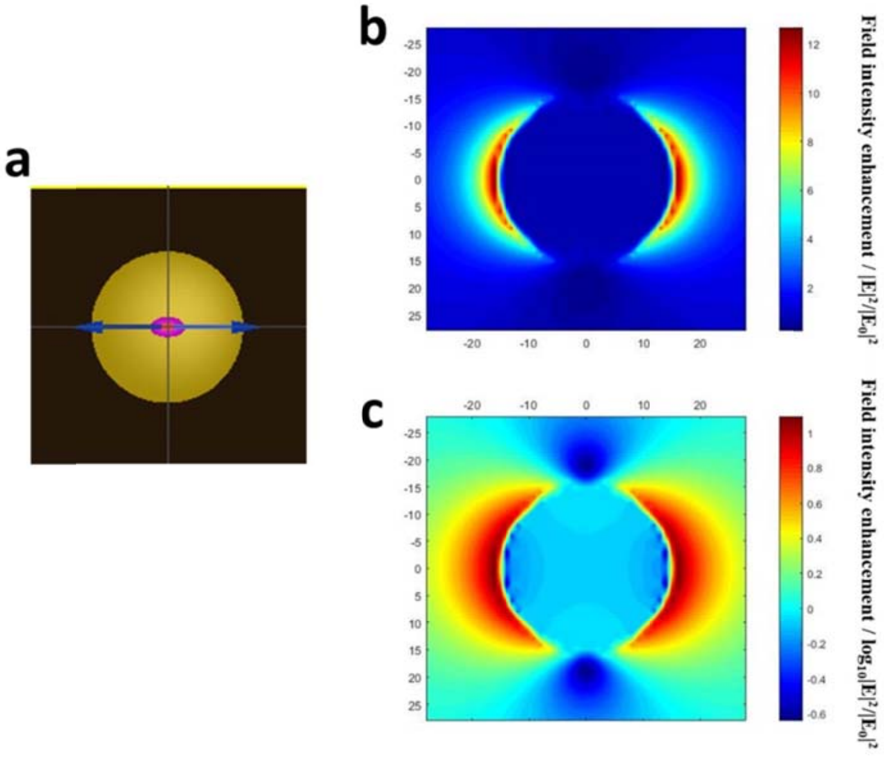

Supplementary Figure 3| FDTD simulation of near-field distribution of single Au nanoparticle exited by visible light: (a) Schematic diagram of simulation model, the pink dot represents the direction of light propagation and the blue arrows represent the polarization of incident light. (b) Simulation result of near-field distribution represent in the form of $|E|^2/|E_0|^2$. (c) Simulation result of near-field distribution represent in the form of $\log_{10}(|E|^2/|E_0|^2)$.

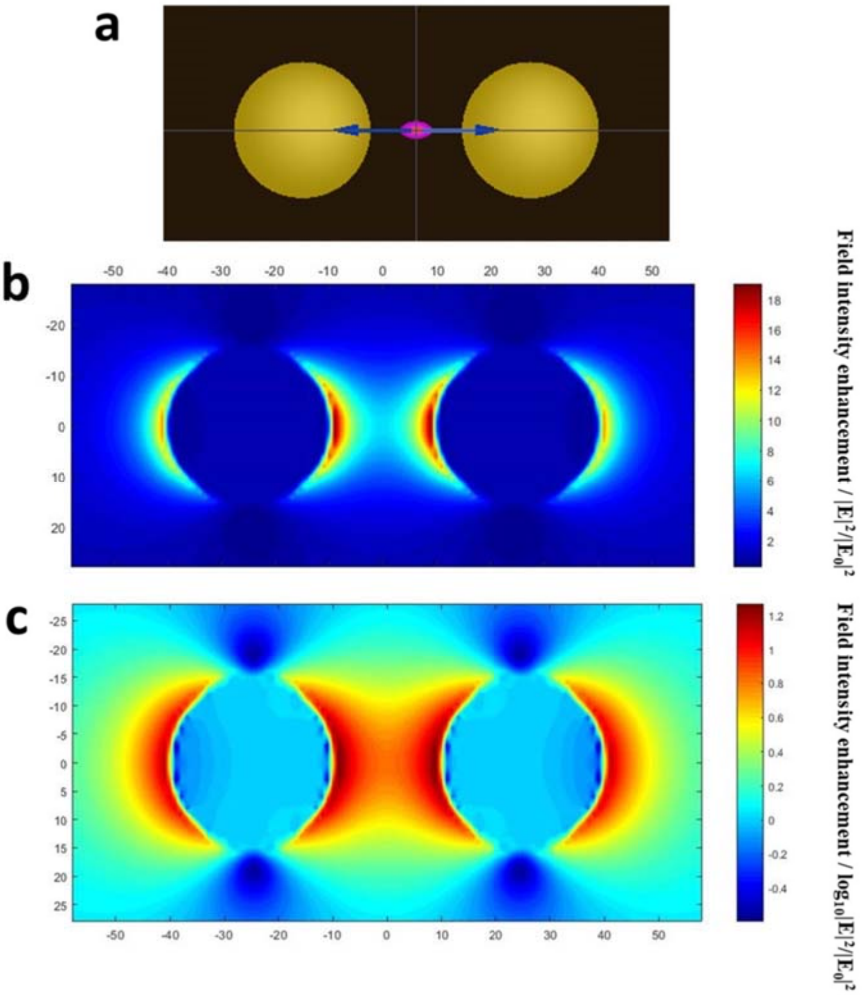

Supplementary Figure 4| FDTD simulation of near-field distribution of two isolated Au nanoparticles exited by visible light: (a) Schematic diagram of simulation model, the pink dot represents the direction of light propagation and the blue arrows represent the polarization of incident light. (b) Simulation result of near-field distribution represent in the form of $|E|^2/|E_0|^2$. (c) Simulation result of near-field distribution represent in the form of $\log_{10}(|E|^2/|E_0|^2)$.

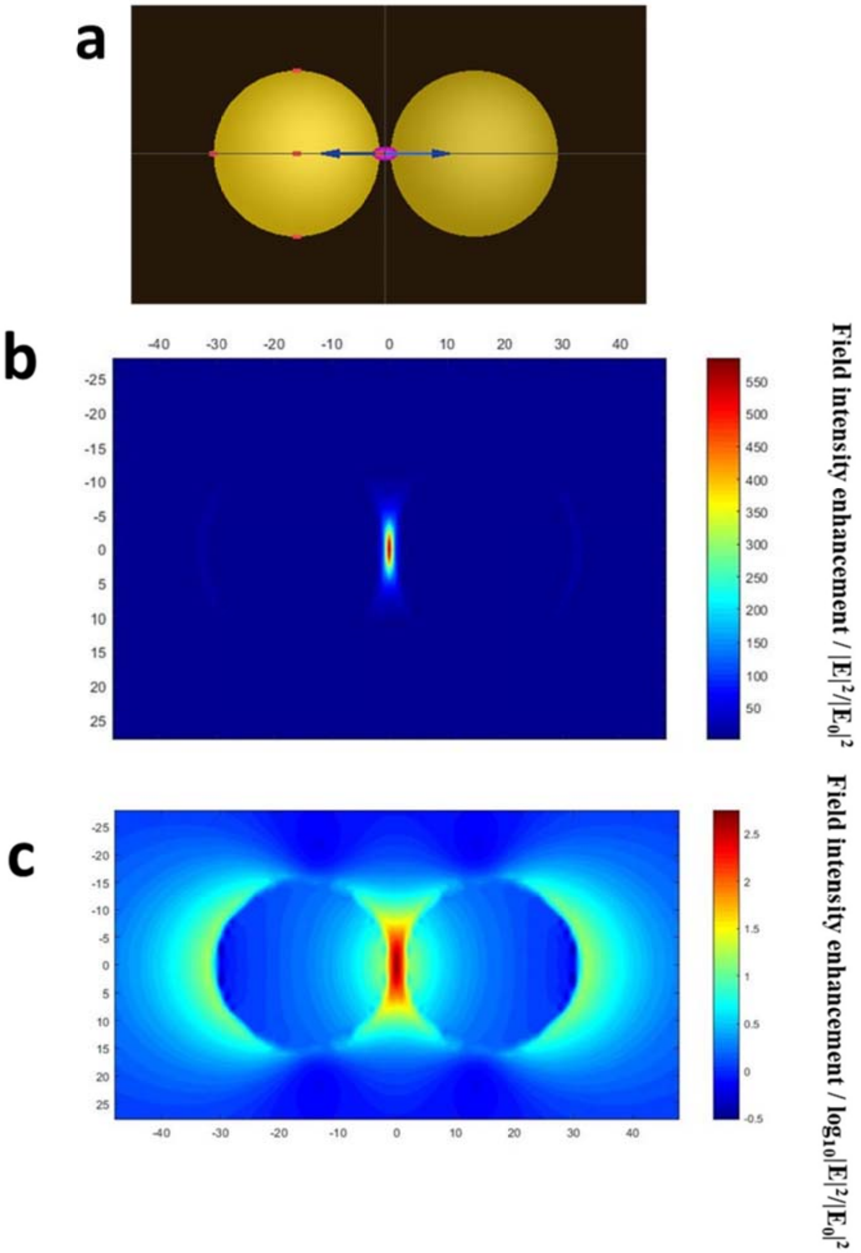

Supplementary Figure 5| FDTD simulation of near-field distribution of coupled Au nanoparticles exited by visible light: (a) Schematic diagram of simulation model, the pink dot represents the direction of light propagation and the blue arrows represent the polarization of incident light. (b) Simulation result of near-field distribution represent in the form of $|E|^2/|E_0|^2$. (c) Simulation result of near-field distribution represent in the form of $\log_{10}(|E|^2/|E_0|^2)$.

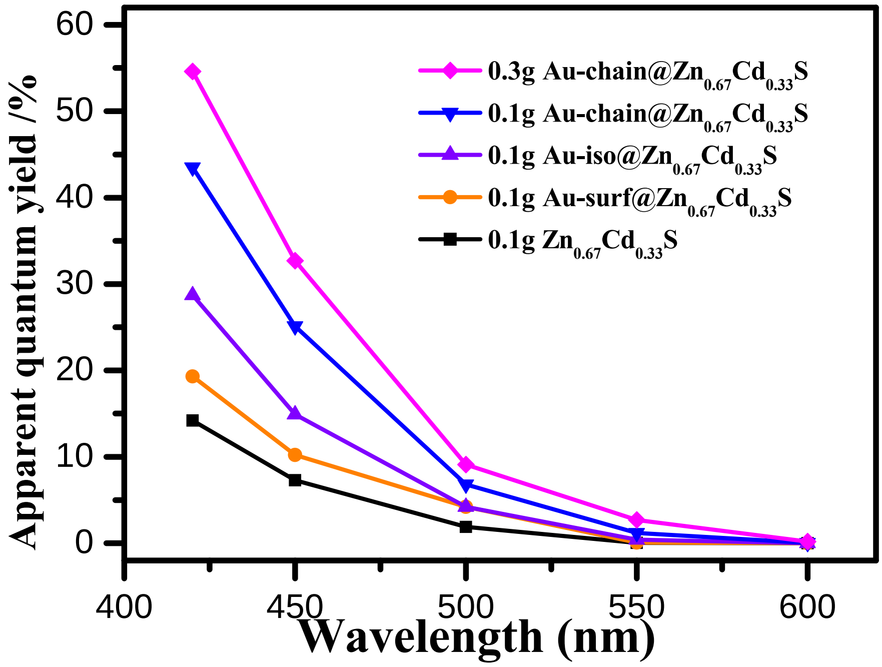

Supplementary Figure 6| Apparent quantum yield of pure $Zn_{0.67}Cd_{0.33}S$ and different Au-containing samples under different wavelength illumination.

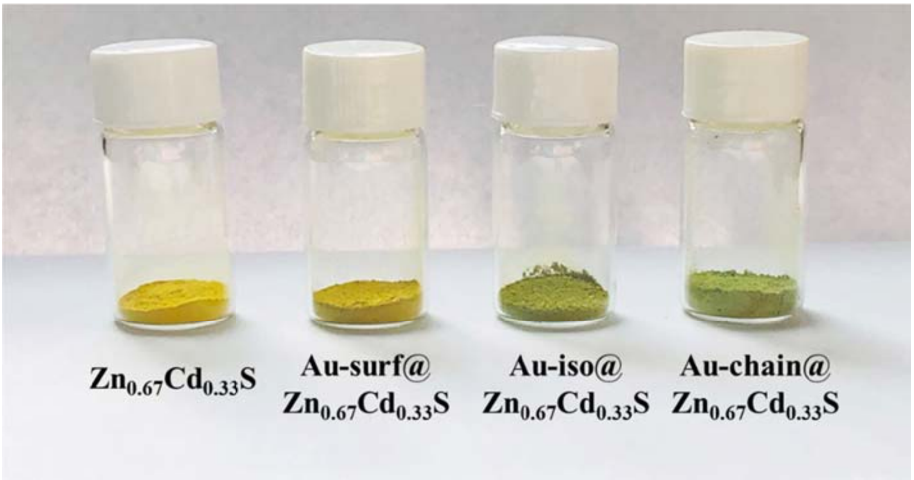

Supplementary Figure 7| Photograph images of pure $Zn_{0.67}Cd_{0.33}S$ and different Au-containing samples.

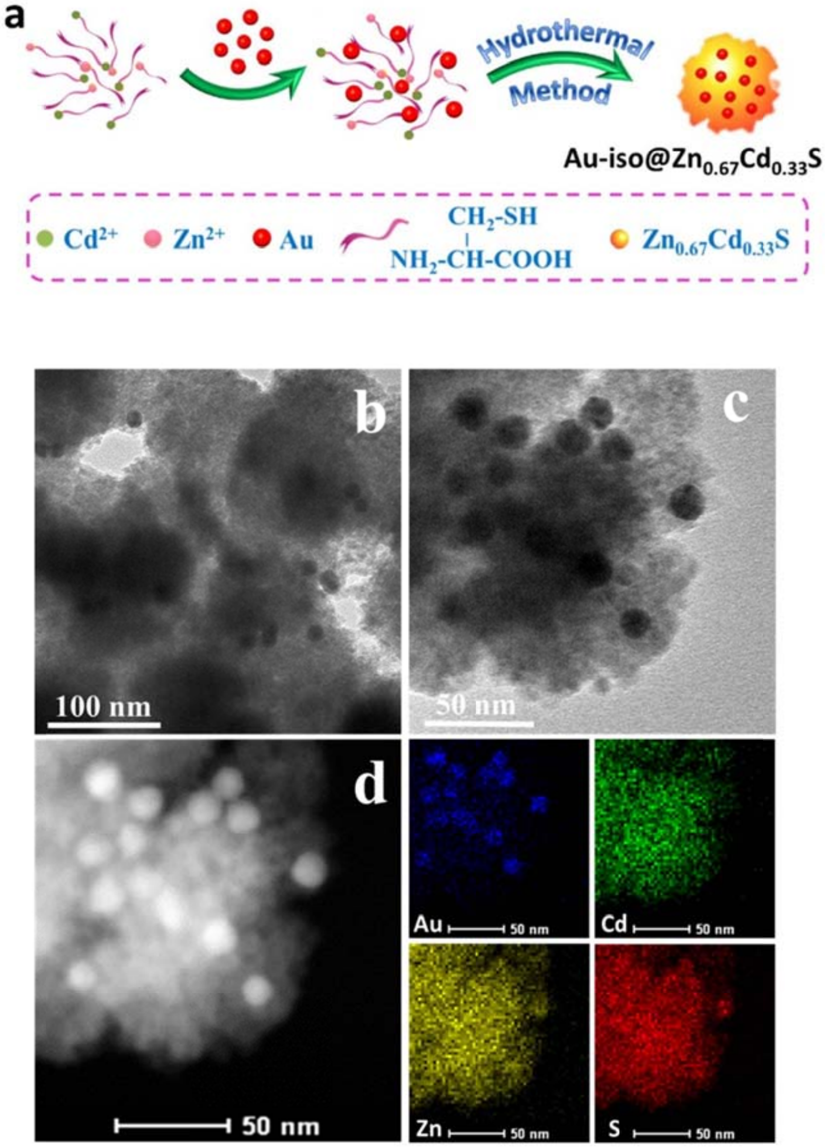

Supplementary Figure 8| (a) Schematic illustration of the preparation process of Au-iso@$Zn_{0.67}Cd_{0.33}S$. (b) and (c) TEM images of Au-iso@$Zn_{0.67}Cd_{0.33}S$. (d) HADDF and EDXS mapping images of Au-iso@$Zn_{0.67}Cd_{0.33}S$.

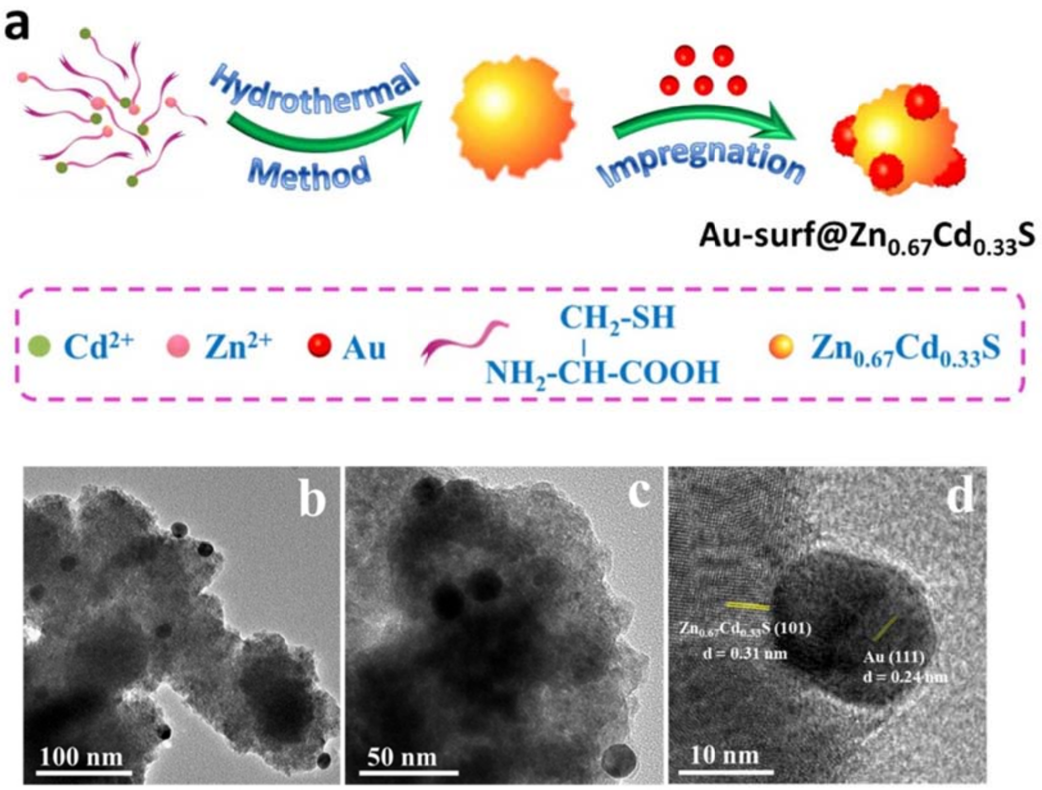

Supplementary Figure 9| (a) Schematic illustration of the preparation process of Au-surf@$Zn_{0.67}Cd_{0.33}S$. (b-d) TEM and HRTEM images of Au nanoparticles on the surface of Au-surf@$Zn_{0.67}Cd_{0.33}S$.

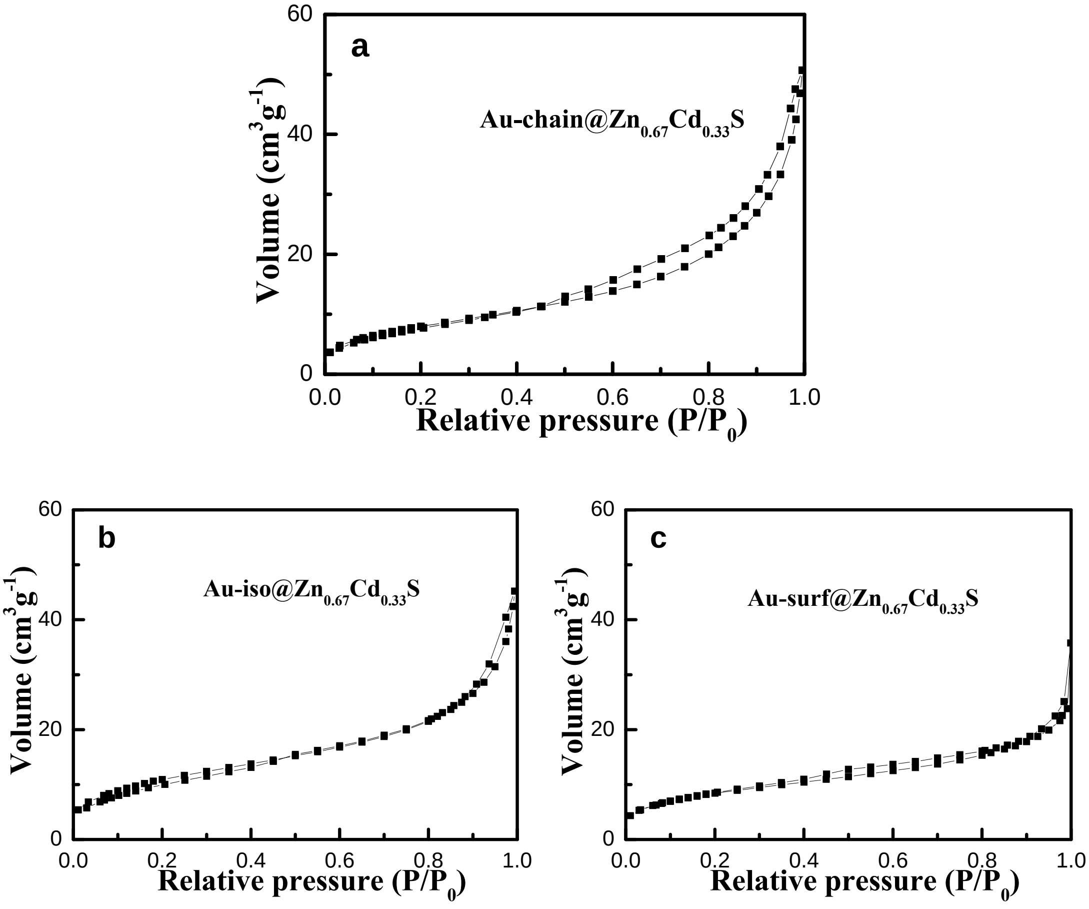

Supplementary Figure 10| N_2 adsorption-desorption isotherms of (a) Au-chain@$Zn_{0.67}Cd_{0.33}S$, (b) Au-iso@$Zn_{0.67}Cd_{0.33}S$ and (c) Au-surf@$Zn_{0.67}Cd_{0.33}S$.

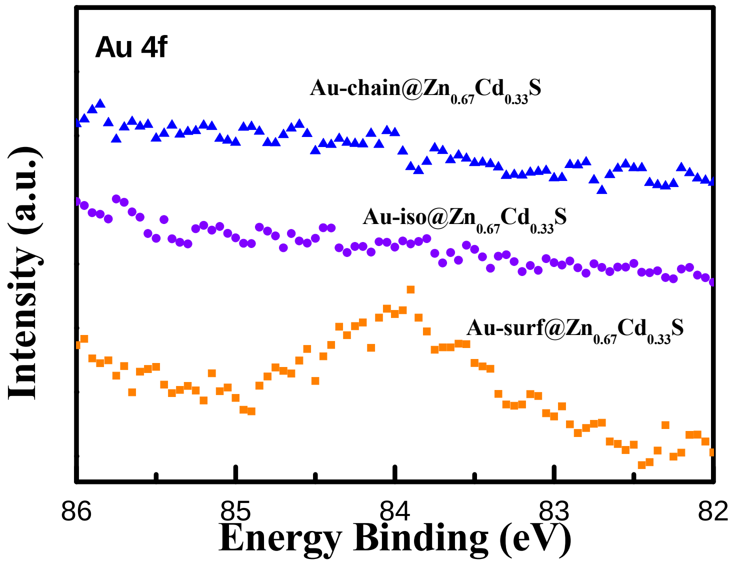

Supplementary Figure 11| Au 4f XPS spectra of three Au-containing samples.

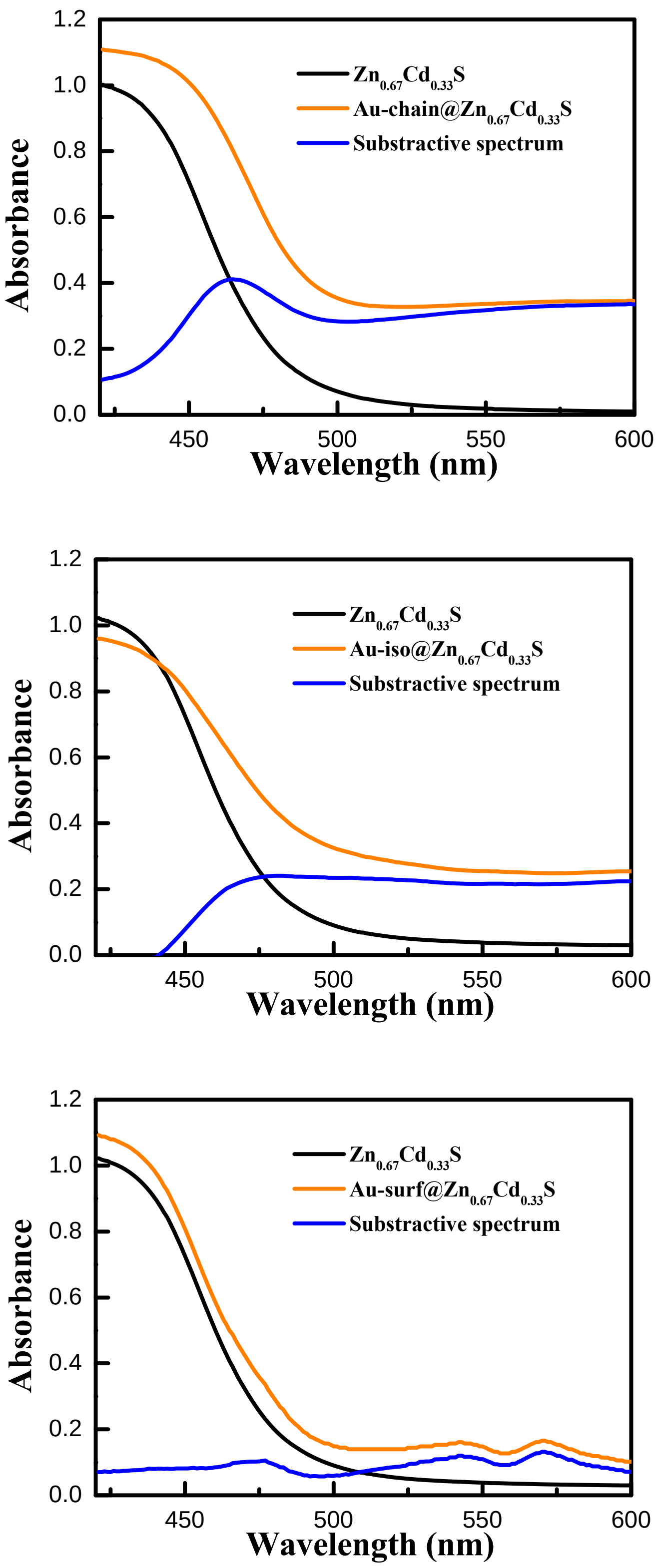

Supplementary Figure 12| Comparison of the contribution of plasmonic-Au to the optical property of $Zn_{0.67}Cd_{0.33}S$ semiconductor.

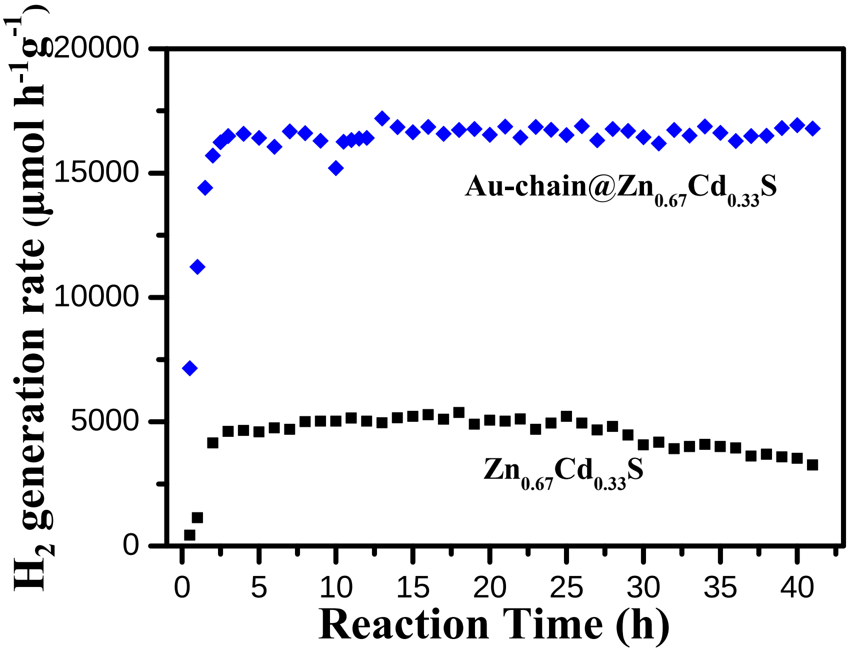

Supplementary Figure 13| Visible-light-driven H_2 evolution rate as a function of time. Reaction condition: 0.1 g photocatalysts in 100 mL Na_2S (0.35 M)-Na_2SO_3 (0.25 M) solution, 300 W Xe-lamp equipped with cut-off filter ($\lambda \geq 420$ nm).

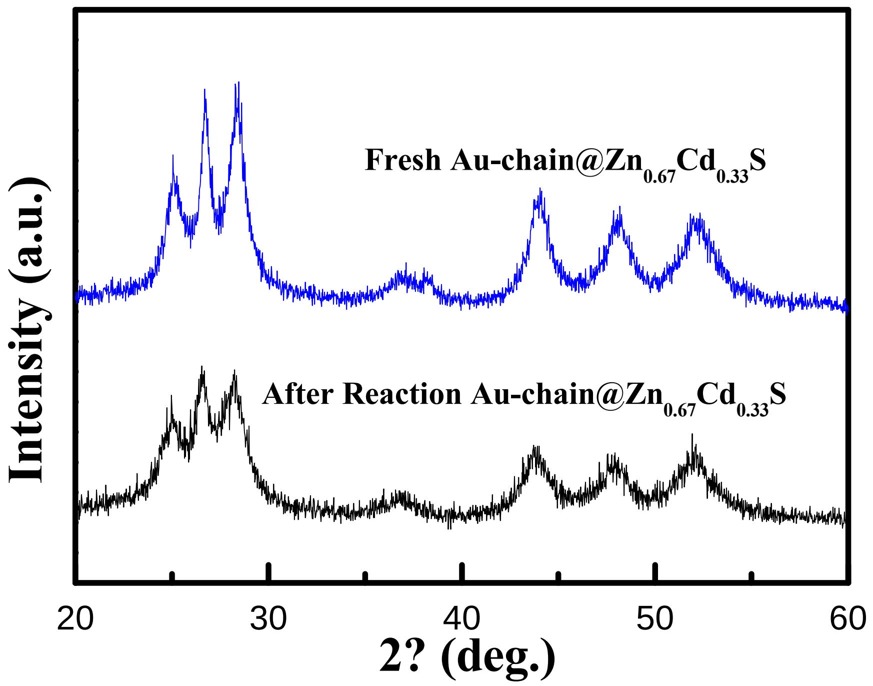

Supplementary Figure 14| XRD patterns of fresh Au-chain@$Zn_{0.67}Cd_{0.33}S$ and used Au-chain@$Zn_{0.67}Cd_{0.33}S$ samples.

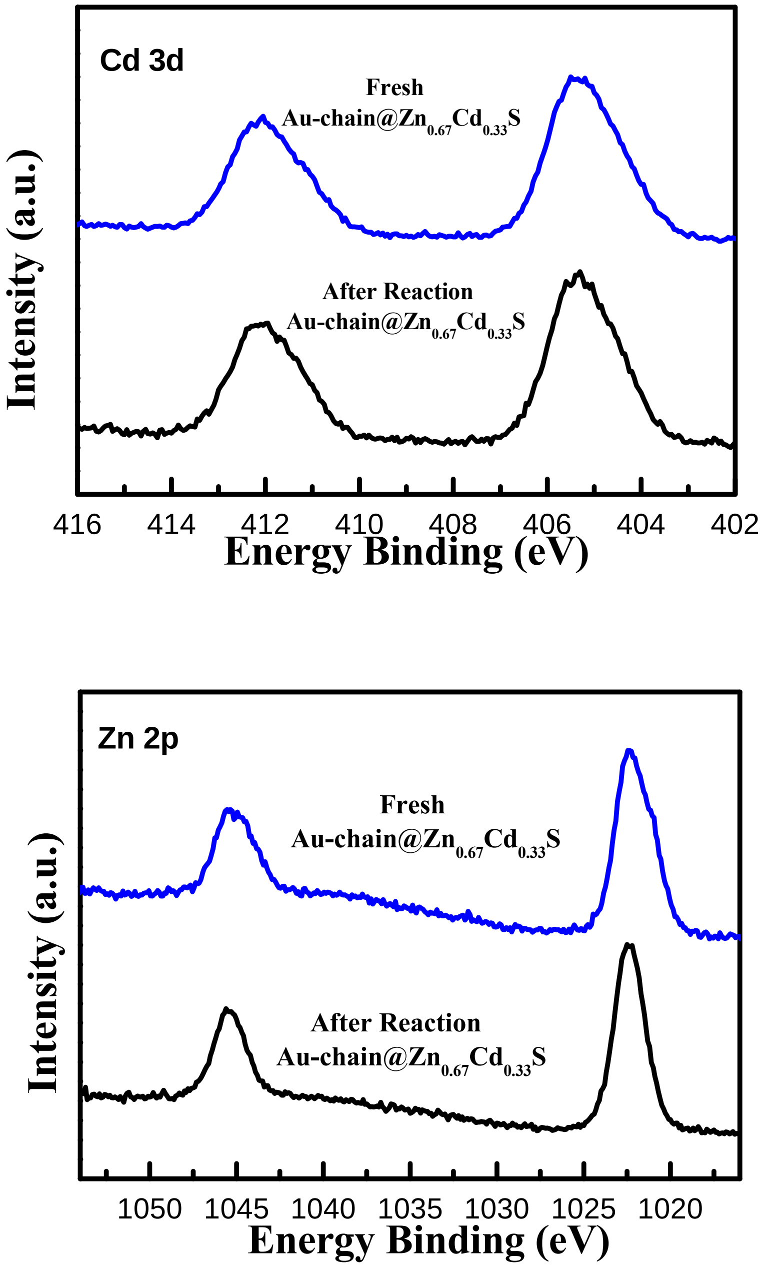

Supplementary Figure 15| Cd 3d and Zn 2p XPS spectra of fresh Au-chain@$Zn_{0.67}Cd_{0.33}S$ and used Au-chain@$Zn_{0.67}Cd_{0.33}S$ samples.

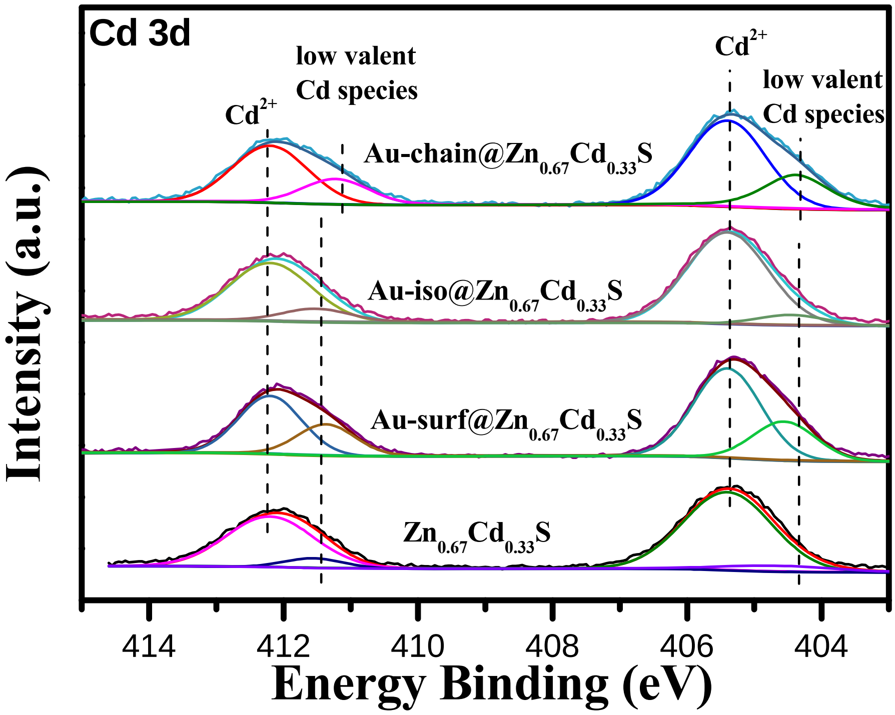

Supplementary Figure 16| Cd 3d XPS spectra of pure $Zn_{0.67}Cd_{0.33}S$ and Au@$Zn_{0.67}Cd_{0.33}S$ with different spatial arrangement of Au nanoparticles.

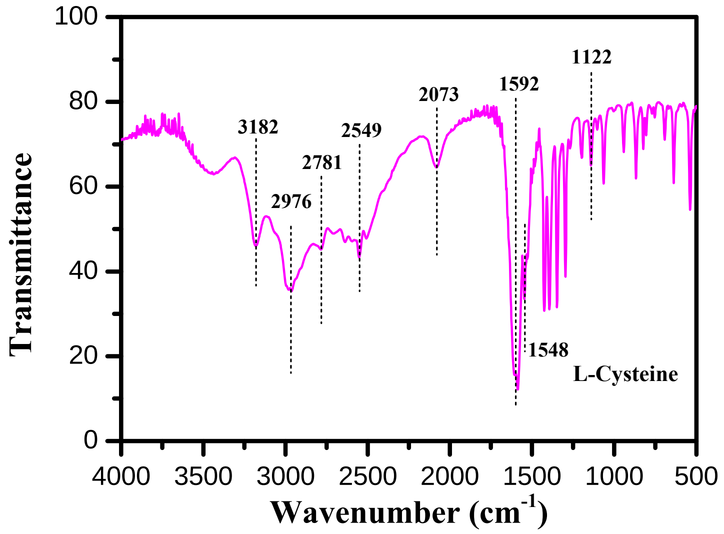

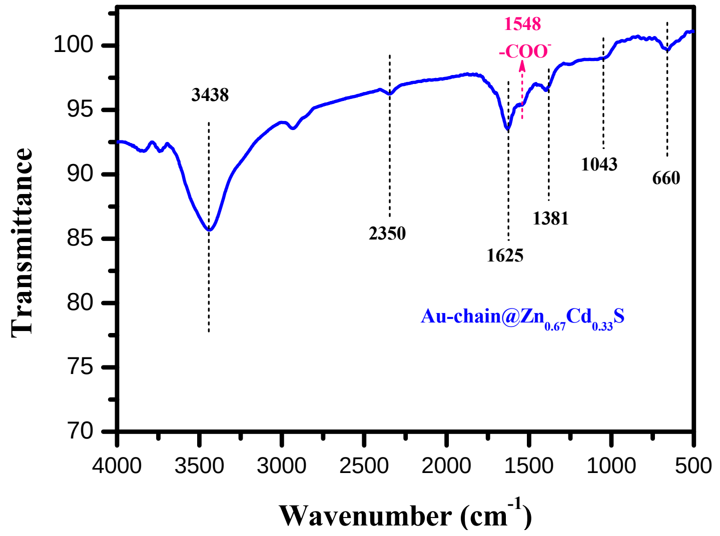

Supplementary Figure 17| FTIR spectra of *L*-cysteine and Au-chain@$Zn_{0.67}Cd_{0.33}S$ samples.

Supplementary Table

Supplementary Table 1 Typical high-performance photocatalysts for hydrogen evolution in recent literatures.

Photocatalyst	Cocatalyst	Light source	H_2 evolution rate ($\mu mol \cdot h^{-1} \cdot g^{-1}$)[a]	QY (%)[b]	Reference
Au@$Zn_{0.67}Cd_{0.33}S$	—	λ≥420nm (300W Xe lamp)	16420	54.6% (420 nm)	This work
$Zn_{0.9}Cd_{0.1}S$[1]	—	λ≥420nm (300W Xe lamp)	4400	—	Chin. J. Catal., 2018, 39, 495-501
ZnO/Pt/$Cd_{0.8}Zn_{0.2}S$[2]	0.5 mol%Pt	UV-visible light (450W Xe lamp)	31200	50.4% (420 nm)	Energy Environ. Sci., 2013, 6, 3589–3594
$Zn_{0.67}Cd_{0.33}S$[3]	—	λ≥420nm (300W Xe lamp)	5604	—	J. Mater. Chem. A, 2016, 4, 13803-13808
$Zn_{0.67}Cd_{0.33}S$[3]	—	λ≥420nm (300W Xe lamp)	7250	—	J. Mater. Chem. A, 2016, 4, 13803-13808
ß-$Ni(OH)_2$/CdS[4]	10 wt%Ni	λ≥420nm (300W Xe lamp)	35000	72% (420 nm)	ACS Catal. 2018, 8, 8726-8738
Au@TiO_2[5]	1wt% Pt	λ≥420nm (300W Xe lamp)	3077	—	ACS Catal. 2018, 8, 4266-4277
C_3N_4[6]	—	λ≥420nm (300W Xe lamp)	13600	12.7% (420 nm)	Adv.Energy, Mater. 2018, 8, 1801139

Continued

Photocatalyst	Cocatalyst	Light source	H_2 evolution rate ($\mu mol \cdot h^{-1} \cdot g^{-1}$)[a]	QY (%)[b]	Reference
Au/ZnO@ZnS[7]	—	320-780 nm (225W Xe lamp)	5690	25.47% (365 nm)	Applied Catal. B, Environmental 2019, 244, 748-780
Fused Sulfone-COF[8]	Pt (8% H_2PtCl_6)	λ≥420nm (300W Xe lamp)	10100	—	Nat. Chem. 2018, 10, 1180-1189
2D-NiO[9]	—	AM 1.5G	10084.6	—	Nat. Commun. 2018, 9, 4036
Co-P/Black P[10]	—	λ≥420nm (300W Xe lamp)	735	42.55% (430 nm 353K)	Nat. Commun. 2018, 9, 1387
$Zn_{0.6}Cd_{0.4}S$[11]	—	λ≥420nm (300W Xe lamp)	5680	—	J. Mater. Chem. A, 2017, 5, 24116-24125.
$Zn_{0.5}Cd_{0.5}S$[12]	10% CoP	AM 1.5G	12175.8	45% (365 nm)	Applied Catal. B, Environmental 2018, 230, 210-219.
$Zn_{0.5}Cd_{0.5}S$[13]	1 wt% $CoPt_3$	λ≥420nm (300W Xe lamp)	2830	—	Int. J. Energy Res. 2016, 40, 1280-1286.
RGO- $Zn_{0.8}Cd_{0.2}S$[14]	—	Solar-simulator (100mW/cm^2)	1824	23.4(420 nm)	Nano Lett. 2012, 12, 4584-4589
CuS- $Zn_{0.17}Cd_{0.83}S$[15]	1% Pt	λ≥420nm (300W Xe lamp)	2463	19.1(420 nm)	Int. J. Hydrogen Energy 2009, 34, 8495-8503

Continued

Photocatalyst	Cocatalyst	Light source	H_2 evolution rate ($\mu mol \cdot h^{-1} \cdot g^{-1}$)[a]	QY (%)[b]	Reference
$Zn_{0.5}Cd_{0.5}S$[16]	3 mol% NiS+ 0.25 wt% RGO	AM 1.5G	7510	20.4% (420 nm)	Adv. Energy Mater. 2014, 4, 1301925
$Zn_{0.5}Cd_{0.5}S$[17]	—	λ≥400nm (300W Xe lamp)	13900	43.0 (425nm)	Energy Environ. Sci. 2011, 4, 1372-1378.
$Zn_{0.5}Cd_{0.5}S$[18]	—	λ≥400nm (350W Xe lamp)	7240	9.6 (420 nm)	ACS Catal. 2013, 3, 882-889
$Zn_{0.5}Cd_{0.5}S$[19]	—	λ≥420nm (300W Xe lamp)	11420	16.9(420 nm)	Small, 2016, 12, 793-801
$Zn_{0.8}Cd_{0.2}S$[20]	0.25% Pt	λ≥420nm (300W Xe lamp)	3200	—	Nanoscale, 2012, 4, 2046-2053
$Zn_{0.4}Cd_{0.6}S$[21]	10 wt% α-Fe_2O_3	λ≥420nm (300W Xe lamp)	5368	11.2% (420 nm)	J. Catal. 2017, 353, 81-88.
$Zn_{0.3}Cd_{0.7}S$[22]	—	λ≥420nm (300W Xe lamp)	3500	—	Catal. Today, 2009, 143, 51-56
$Zn_{0.56}Cd_{0.44}S$[23]	—	λ≥420nm (500W Xe lamp)	2640	—	Int. J. Hydrogen Energy 2010, 35, 19-25

Continued

Photocatalyst	Cocatalyst	Light source	H_2 evolution rate ($\mu mol \cdot h^{-1} \cdot g^{-1}$)[a]	QY (%)[b]	Reference
$Zn_{0.8}Cd_{0.2}S$[24]	—	420nm≤λ≤780nm (300W Xe lamp)	3430	16.2(420 nm)	J. Colloid Interface Sci. 2016, 467, 97-104
$Zn_{0.5}Cd_{0.5}S$[25]	—	150W Xe lamp	2000	—	Ind. Eng. Chem. Res. 2010, 49, 6854-6861
$Zn_{0.5}Cd_{0.5}S$[26]	—	300W Hg lamp	1019	1.29(420 nm)	Int. J. Hydrogen Energy 2014, 39, 1630-1639
$Zn_{0.17}Cd_{0.83}S$-gC_3N_4[27]	—	λ≥420nm (300W Xe lamp)	—	37(420 nm)	Int. J. Hydrogen Energy 2015, 40, 7546-7552
$Zn_{0.5}Cd_{0.5}S$[28]	1.5 wt% MoS_2+ 3 wt% RGO	AM 1.5G	7700	—	ACS Applied Mater. Interfaces 2016, 8, 2928-2934.
$Cu_{1.94}S$-$Zn_{0.77}Cd_{0.23}S$[29]	5% Pt	λ≥420nm (300W Xe lamp)	13533	26.4 (420 nm)	J. Am. Chem. Soc. 2016, 138, 4286-4289

[a] H_2 evolution rate calculated based on the optimized activity in the corresponding literature.

[b] Apparent quantum yield.

Supplementary Methods

Supplementary Method 1| Preparation of isolated Au nanoparticles embedded into $Zn_{0.67}Cd_{0.33}S$ (denoted as Au-iso@$Zn_{0.67}Cd_{0.33}S$).

Au-iso@$Zn_{0.67}Cd_{0.33}S$ was prepared with the same procedure and composition as the method of Au-chain@$Zn_xCd_{1-x}S$ photocatalyst except changing diluted condition before transferring into Teflon-lined stainless-steel autoclaves. Typically, the cysteine- Zn^{2+}/Cd^{2+}-coupled Au colloids were diluted to a total volume of 30 mL with deionized water, and transferred into 40 mL Teflon-lined stainless-steel autoclaves. The autoclaves were maintained at 130 °C for 6 h and then cooled to room temperature naturally. After filtration and wash process, Au-iso@$Zn_xCd_{1-x}S$ composite can be obtained in an oven at 80 °C for 12 h.

Supplementary Method 2| Preparation of Au nanoparticles loaded on the surface of $Zn_{0.67}Cd_{0.33}S$ (denoted as Au-surf@$Zn_{0.67}Cd_{0.33}S$).

Au-surf@$Zn_{0.67}Cd_{0.33}S$ was prepared by an impregnation method. Briefly, 0.2 g of pre-obtained $Zn_{0.67}Cd_{0.33}S$ was dispersed in a certain amount of Au-Cit colloids (0.25 mM, 40 mL) and the mixture was stirred at room temperature for 4 h. Then the mixture was heated at 60 °C in open air to remove water and other volatiles. After that, the products were fully dried at 80 °C in an oven to obtain the final product.

Supplementary References

1. Zhao, H. et al. Biomolecule-assisted, cost-effective synthesis of a $Zn_{0.9}Cd_{0.1}S$ solid solution for efficient photocatalytic hydrogen production under visible light. *Chin. J. Catal.* **39**, 495-501 (2018).
2. Lingampalli, S.R., Gautam, U.K. & Rao, C.N.R. Highly efficient photocatalytic hydrogen generation by solution-processed ZnO/Pt/CdS, ZnO/Pt/$Cd_{1-x}Zn_xS$ and ZnO/Pt/$CdS_{1-x}Se_x$ hybrid nanostructures. *Energy Environ. Sci.* **6**, 3589-3594 (2013).
3. Yu, G. et al. A highly active cocatalyst-free semiconductor photocatalyst for visible-light-driven hydrogen evolution: synergistic effect of surface defects and spatial bandgap engineering. *J. Mater. Chem. A* **4**, 13803-13808 (2016).
4. Vamvasakis, I. et al. Visible-Light Photocatalytic H_2 Production Activity of *β*-$Ni(OH)_2$-Modified CdS Mesoporous Nanoheterojunction Networks. *ACS Catal.* **8**, 8726-8738 (2018).
5. Zhao, M. et al. Fabricating a Au@TiO_2 Plasmonic System To Elucidate Alkali-Induced Enhancement of Photocatalytic H_2 Evolution: Surface Potential Shift or Methanol Oxidation Acceleration? *ACS Catal.* **8**, 4266-4277 (2018).
6. Zhang, Y. et al. Leaf-Mosaic-Inspired Vine-Like Graphitic Carbon Nitride Showing High Light Absorption and Efficient Photocatalytic Hydrogen Evolution. *Adv. Energy Mater.* **8**, 1801139 (2018).
7. Ma, D. et al. Au decorated hollow ZnO@ZnS heterostructure for enhanced photocatalytic hydrogen evolution: The insight into the roles of hollow channel and Au nanoparticles. *Appl. Catal., B* **244**, 748-757 (2019).
8. Wang, X. et al. Sulfone-containing covalent organic frameworks for photocatalytic hydrogen evolution from water. *Nat. Chem.* **10**, 1180-1189 (2018).
9. Lin, Z., Du, C., Yan, B., Wang, C. & Yang, G. Two-dimensional amorphous NiO as a plasmonic photocatalyst for solar H_2 evolution. *Nat. Commun.* **9**, 4036 (2018).
10. Ikeda, T. et al. Srf destabilizes cellular identity by suppressing cell-type-specific

gene expression programs. *Nat. Commun.* **9**, 1387 (2018).

11. Chen, J., Chen, J. & Li, Y. Hollow ZnCdS dodecahedral cages for highly efficient visible-light-driven hydrogen generation. *J. Mater. Chem. A* **5**, 24116-24125 (2017).
12. Wang, P. et al. Cobalt phosphide nanowires as efficient co-catalyst for photocatalytic hydrogen evolution over $Zn_{0.5}Cd_{0.5}S$. *Appl. Catal., B* **230**, 210-219 (2018).
13. Wang, H. et al. $CoPt_x$-loaded $Zn_{0.5}Cd_{0.5}S$ nanocomposites for enhanced visible light photocatalytic H_2 production. *Int. J. Energy Res.* **40**, 1280-1286 (2016).
14. Zhang, J., Yu, J., Jaroniec, M. & Gong, J.R. Noble Metal-Free Reduced Graphene Oxide-$Zn_xCd_{1-x}S$ Nanocomposite with Enhanced Solar Photocatalytic H_2-Production Performance. *Nano Lett.* **12**, 4584-4589 (2012).
15. Zhang, W. & Xu, R. Surface engineered active photocatalysts without noble metals: CuS–$Zn_xCd_{1-x}S$ nanospheres by one-step synthesis. *Int. J. Hydrogen Energy* **34**, 8495-8503 (2009).
16. Zhang, J., Qi, L., Ran, J., Yu, J. & Qiao, S.Z. Ternary NiS/$Zn_xCd_{1-x}S$/Reduced Graphene Oxide Nanocomposites for Enhanced Solar Photocatalytic H_2-Production Activity. *Adv. Energy Mater.* **4**, 1301925 (2014).
17. Liu, M., Wang, L., Lu, G., Yao, X. & Guo, L. Twins in $Cd_{1-x}Zn_xS$ solid solution: Highly efficient photocatalyst for hydrogen generation from water. *Energy Environ. Sci.* **4**, 1372-1378 (2011).
18. Li, Q. et al. $Zn_{1-x}Cd_xS$ Solid Solutions with Controlled Bandgap and Enhanced Visible-Light Photocatalytic H_2-Production Activity. *ACS Catal.* **3**, 882-889 (2013).
19. Zhang, X. et al. Surface Defects Enhanced Visible Light Photocatalytic H_2 Production for Zn-Cd-S Solid Solution. *Small* **12**, 793-801 (2016).
20. Wang, D.-H., Wang, L. & Xu, A.-W. Room-temperature synthesis of $Zn_{0.80}Cd_{0.20}S$ solid solution with a high visible-light photocatalytic activity for hydrogen evolution. *Nanoscale* **4**, 2046-2053 (2012).

21. Imran, M., Yousaf, A.B., Kasak, P., Zeb, A. & Zaidi, S.J. Highly efficient sustainable photocatalytic Z-scheme hydrogen production from an α-Fe_2O_3 engineered ZnCdS heterostructure. *J. Catal.* **353**, 81-88 (2017).
22. del Valle, F. et al. Influence of Zn concentration in the activity of $Cd_{1-x}Zn_xS$ solid solutions for water splitting under visible light. *Catal. Today* **143**, 51-56 (2009).
23. Wang, L. et al. Enhanced photocatalytic hydrogen evolution under visible light over $Cd_{1-x}Zn_xS$ solid solution with cubic zinc blend phase. *Int. J. Hydrogen Energy* **35**, 19-25 (2010).
24. Zhou, Y. et al. Mesoporous $Cd_{1-x}Zn_xS$ microspheres with tunable bandgap and high specific surface areas for enhanced visible-light-driven hydrogen generation. *J. Colloid Interface Sci.* **467**, 97-104 (2016).
25. Villoria, J.A., Navarro Yerga, R.M., Al-Zahrani, S.M. & Fierro, J.L.G. Photocatalytic Hydrogen Production on $Cd_{1-x}Zn_xS$ Solid Solutions under Visible Light: Influence of Thermal Treatment. *Ind. Eng. Chem. Res.* **49**, 6854-6861 (2010).
26. Chan, C.-C. et al. Efficient and stable photocatalytic hydrogen production from water splitting over $Zn_xCd_{1-x}S$ solid solutions under visible light irradiation. *Int. J. Hydrogen Energy* **39**, 1630-1639 (2014).
27. Wang, X., Chen, J., Guan, X. & Guo, L. Enhanced efficiency and stability for visible light driven water splitting hydrogen production over $Cd_{0.5}Zn_{0.5}S$/g-C_3N_4 composite photocatalyst. *Int. J. Hydrogen Energy* **40**, 7546-7552 (2015).
28. Guo, S.N., Min, Y.L., Fan, J.C. & Xu, Q.J. Stabilizing and Improving Solar H_2 Generation from $Zn_{0.5}Cd_{0.5}S$ Nanorods@MoS_2/RGO Hybrids via Dual Charge Transfer Pathway. *ACS Appl. Mater. Interfaces* **8**, 2928-2934 (2016).
29. Chen, Y. et al. Synergetic Integration of $Cu_{1.94}S$–$Zn_xCd_{1-x}S$ Heteronanorods for Enhanced Visible-Light-Driven Photocatalytic Hydrogen Production. *J. Am. Chem. Soc.* **138**, 4286-4289 (2016).
